# HIPPs of all trades: insights from pathogens on the function of HMA proteins at plasmodesmata

**DOI:** 10.1093/jxb/eraf234

**Published:** 2025-05-28

**Authors:** Emma K Turley, Christine Faulkner

**Affiliations:** Cell and Developmental Biology, John Innes Centre, Norwich Research Park, Norwich NR4 7UH, UK; Cell and Developmental Biology, John Innes Centre, Norwich Research Park, Norwich NR4 7UH, UK; University of Wisconsin, Madison, USA

**Keywords:** Effector, heavy metal, HIPP, HMA, HPP, metallochaperone, NLR, pathogen, plasmodesmata

## Abstract

Within plant cell walls, plasmodesmal channels harbour unique collections of proteins to maintain their structure and facilitate dynamic regulation of cell-to-cell connectivity. Proteomic surveys, combined with evidence from confocal microscopy, have identified heavy metal-associated (HMA) domain-containing proteins as residents at plasmodesmata; however, the functional relevance of this localization is currently unknown. Although HMA domains themselves are present in all kingdoms of life, in plants they can be found in three main families: HPPs, HIPPs, and P_1B_-type ATPases. Within the last decade, HPPs and HIPPs have emerged as frequent host targets of pathogen-derived molecules, including secreted effectors from bacteria, fungi, and oomycetes, and a viral movement protein. The seemingly conserved targeting of HMA domains throughout distantly related pathosystems suggests that these proteins could play integral roles in plant immunity. This is corroborated by observations of HMA-like domains being integrated into plant immune receptors, enabling direct binding of effectors to activate downstream signalling, as well as genetic evidence highlighting the influence of HPPs and HIPPs on disease susceptibility. Drawing especially from plant pathology studies, we speculate about the potential metallochaperone and signalling functions of these enigmatic plasmodesmal components.

## Teaser

We explore the function of plant heavy metal-associated domain-containing proteins and speculate about their functions at plasmodesmata by drawing from plant–pathogen interaction studies.

## Introduction

As key components of the internal communication system of plants, plasmodesmata directly connect the cytoplasm, plasma membrane, and endoplasmic reticulum (ER) of adjacent cells, enabling transport of proteins, small RNAs, phytohormones, and nutrients. The ultrastructure of an individual plasmodesma is typically represented as a plasma membrane-lined pore containing a cytoplasmic sleeve and appressed strand of ER called the desmotubule (Sager and Lee, 2018).

In comparison with the wider plasma membrane, plasmodesmata harbour unique communities of proteins. Previously, meticulous fractionation and MS were used to enrich for cell wall and plasmodesmal proteins in plants and algae (e.g. see Faulkner *et al*., 2005; Bayer *et al*., 2006). This led to the identification of key regulators of plasmodesmal function such as β-1,3-glucanases, which degrade plasmodesmata-associated callose to enhance intercellular trafficking (Levy *et al*., 2007; Benitez-Alfonso *et al*., 2013), and PLASMODESMATA-LOCATED PROTEINs (PDLPs) that function in localized signalling cascades (Thomas *et al*., 2008; Tee *et al*., 2023).

More recently, armed with novel and previously published proteomes from divergent species, namely *Arabidopsis thaliana* (from hereon Arabidopsis), *Populus trichocarpa*, and *Physcomitrium patens*, Johnston *et al*. (2023) identified conserved protein orthogroups represented in plasmodesmata. One such orthogroup consisted of HEAVY METAL-ASSOCIATED ISOPRENYLATED PLANT PROTEINs (HIPPs), which are prevalent throughout vascular plant genomes and defined as having one or two heavy metal-associated (HMA) domains, and a C-terminal isoprenylation motif (Barth *et al*., 2009; de Abreu-Neto *et al*., 2013).

So far, 11 HIPP orthologues have emerged in plasmodesmal proteomes (reviewed by Barr *et al*., 2023), and the subcellular localizations of three of these (AtHIPP01 and AtHIPP07 from Arabidopsis, and NbHIPP26 from *Nicotiana benthamiana*) were confirmed (Cowan *et al*., 2018; Guo *et al*., 2021). Additionally, OsHIPP17, OsHIPP19, and OsHIPP20 from rice (*Oryza sativa*) and HvHIPP43 from barley (*Hordeum vulgare*) were identified in different contexts and subsequently observed to localize to plasmodesmata (Oikawa *et al*., 2024; Were *et al*., 2025). Although lacking an isoprenylation motif, HMA PLANT PROTEIN (HPP) relatives of HIPPs were also detected in plasmodesmal proteomes from Arabidopsis (Fernandez-Calvino *et al*., 2011), *N. benthamiana* (Park *et al*., 2017), and *P. patens* (Gombos *et al*., 2023). Despite the growing list of plasmodesmal HMA domain-containing proteins, exactly why they accumulate here remains a mystery.

Independent of their appearance in plasmodesmata, HIPPs and HPPs are common players in plant–pathogen interactions, being frequently targeted by pathogen-derived proteins and identified as regulators of infection success. To explain this phenomenon, we hypothesize that these proteins might play important and potentially diverse roles in plant immunity that evolutionarily distant pathogens have convergently evolved to manipulate. In this review, we explore the known functions of HMA domain-containing proteins, drawing especially from plant pathology studies to provide clues as to the endogenous functions of these enigmatic proteins at plasmodesmata.

## A historical perspective: what makes a HMA domain?

Perhaps the most obvious hint towards HMA domain function can be found in the name. Bull and Cox (1994) coined the term HMA to describe a collection of sequences from humans and bacteria, each possessing a conserved CXXC motif to facilitate metal ion co-ordination. These sequences included the Menkes and Wilson disease proteins from humans (ATP7A and ATP7B, respectively), which are P_1B_-type ATPases required to shuttle copper (Cu) across intracellular membranes within the secretory pathway (Tanzi *et al*., 1993; Vulpe *et al*., 1993). The characteristic 3D structure of a HMA domain was first described by Eriksson and Sahlman (1993), when the NMR structure of MerP, a periplasmic mercury (Hg)-binding protein from *Pseudomonas aeruginosa*, revealed a βαββαβ secondary structure, comprising an antiparallel β-sheet with two α-helices folded against it. A subsequent NMR structure of the fourth metal-binding domain of ATP7A (Gitschier *et al*., 1998) and the crystal structure of the yeast (*Saccharomyces cerevisiae*) metallochaperone, antioxidant protein 1 (ATX1) (Rosenzweig *et al*., 1999, Fig. 1), each displayed homologous αβ-sandwich folds, now recognized (alongside the CXXC motif) as a defining feature of HMA domains (InterPro: IPR006121).

**Fig. 1.**
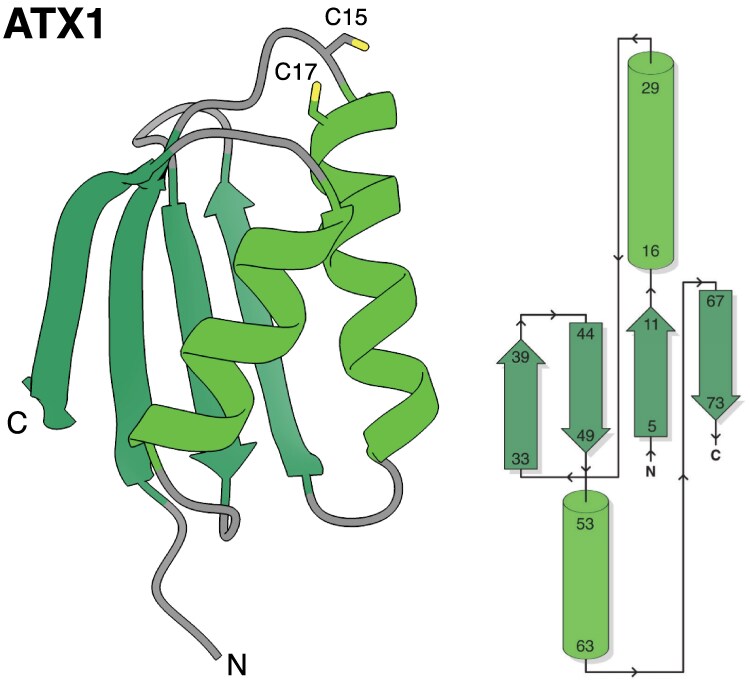
HMA domain structure and topology. Crystal structure of ATX1 from *S. cerevisiae* (1CC8) (Rosenzweig *et al*., 1999), demonstrating a typical βαββαβ topology. Cys15 and Cys17 of the metal-binding motif (MTCSGC) are annotated. A topology diagram was drawn with the aid of PDBsum (Laskowski *et al*., 2018).

*In vitro* studies indicated that prototypical HMA domains were capable of direct heavy metal binding. Further structural characterization of MerP revealed a Hg(II) ion co-ordinated between the two Cys residues of the CXXC motif, forming approximately linear co-ordinate bonds (Steele and Opella, 1997). On the other hand, ATP7A, ATX1, and its human homologue HAH1 (later named ATOX1) bound Cu(I) *in vitro* (Lutsenko *et al*., 1997; Pufahl *et al*., 1997; Hung *et al*., 1998).

Across kingdoms, HMA domain-containing proteins were initially characterized as metallochaperones: proteins that shuttle metal ions intracellularly to other metalloproteins. For instance, homologous Cu transport pathways in yeast, humans, and Arabidopsis each employ a cytosolic HMA domain (ATX1, ATOX1, or AtATX1, respectively) to deliver imported Cu(I) to a HMA domain-containing ATPase embedded within post-Golgi vesicles (CCC2, ATP7A/B, or RAN1, respectively) (Yuan *et al*., 1995; Klomp *et al*., 1997; Pufahl *et al*., 1997; Puig *et al*., 2007; Muller and Klomp, 2009). Two more Cu chaperones with HMA domains have been characterized in Arabidopsis: AtCCH (Cu chaperone) and AtCCS, a Cu chaperone for Cu–zinc (Zn) superoxide dismutase (Cu/Zn-SOD) (Mira *et al*., 2001; Abdel-Ghany *et al*., 2005; Chu *et al*., 2005). Beyond this handful of Cu-binding examples, there is a growing body of evidence to support roles for plant HMA domains in regulation of heavy metal tolerance (see ‘What microbes aren’t telling us: HPP/HIPP function in heavy metal tolerance’). However, numerous domains annotated as ‘HMA’ in plants lack the defining CXXC motif (and are thus referred to from hereon as ‘HMA-like’ domains), suggesting that metals cannot be bound unless via an alternative mechanism. Considered alongside the diversity and expansion of HMA domain-containing families in plants (see below and Table 1), this suggests the possibility of additional functions transcending metal homeostasis.

**Table 1.** Number of *HIPP* and *HPP* genes identified in surveys of vascular plant genomes to date*^a^*

Species	*HIPP* genes	*HPP* genes	Reference
*Aegilops tauschii*	40	nr	H. Zhang *et al*. (2020)
*Arabidopsis thaliana*	47	21	de Abreu-Neto *et al*. (2013); Li *et al*. (2020)
*Brachypodium distachyon*	40	nr	H. Zhang *et al*. (2020)
*Camellia sinensis*	38	9	Wei *et al*. (2023) *^b^*
*Chenopodium quinoa*	34	nr	Sun *et al*. (2022)
*Citrus sinensis*	26	nr	Huang *et al*. (2024)
*Fagopyrum tataricum*	13	36	Ye *et al*. (2022) *^c^*
*Haynaldia villosa*	13	nr	H. Zhang *et al*. (2020)
*Hordeum vulgare*	33	nr	H. Zhang *et al*. (2020)
*Oryza sativa*	59	7	de Abreu-Neto *et al*. (2013)
*Populus trichocarpa*	74	10	de Abreu-Neto *et al*. (2013)
*Setaria italica*	52	3	de Abreu-Neto *et al*. (2013)
*Selaginella moellendorffii*	5	4	de Abreu-Neto *et al*. (2013)
*Solanum lycopersicum*	28	6	Xu *et al*. (2024) *^d^*
*Triticum aestivum*	114	nr	H. Zhang *et al*. (2020)
*Triticum dicoccoides*	58	nr	H. Zhang *et al*. (2020)
*Triticum urartu*	33	nr	H. Zhang *et al*. (2020)

^*a*^nr, not reported.

^*b*^A total of 56 genes were assigned the name *HIPP*. However, this list included nine genes encoding HPPs and nine genes encoding putative ATPases.

^*c*^ *HPP* genes were assigned the name *FtATX1*.

^*d*^A total of 34 genes were assigned the name *HIPP*. However, this list included six genes encoding HPPs.

Plant proteins sporting HMA or HMA-like domains (from hereon HMA proteins) can be divided into three broad families: HPPs, HIPPs, and P_1B_-type ATPases. On rare occasions, HMA-like domains can also be found integrated within plant immune receptors (see ‘HMA domains as bait for effectors’). HIPPs, initially called *Arabidopsis thaliana* farnesylated proteins (ATFPs), or *Glycine max* farnesylated proteins (GMFPs), were first described by Dykema *et al*. (1999), following their identification in an *in vitro* screen for cDNAs encoding isoprenylated proteins (Crowell *et al*., 1996). These first HIPPs (ATFP2, 3, 6, and GMFP5, 7) were noted to possess both metal-binding (M/LXCXXC) and isoprenylation (CaaX) motifs. Later BLAST-based searches uncovered a 45 member Arabidopsis HIPP family, organized into five clades (Barth *et al*., 2009; Tehseen *et al*., 2010; de Abreu-Neto *et al*., 2013). In addition, a 22 member Arabidopsis HPP family was described, including metallochaperones AtATX1, AtCCS, and AtCCH (Tehseen *et al*., 2010; de Abreu-Neto *et al*., 2013), the members of which lack an isoprenylation motif. Subsequently, using a hidden Markov model, Li *et al*. (2020) identified a further two HIPPs (AtHMP09 and AtHMP35, for heavy metal-associated protein) and two HPPs (AtHMP18 and AtHMP28), bring the total number of family members to 47 and 24, respectively.

HMA domain-containing P_1B_-type ATPases constitute a smaller family of just eight proteins in Arabidopsis, named HMA1–HMA8 (for heavy metal ATPase, not to be confused with heavy metal-associated), only four of which possess at least one canonical HMA domain: HMA5, HMA6/PAA1, HMA7/RAN1, and HMA8/PAA2 (Williams and Mills, 2005). These pumps utilize energy from ATP hydrolysis to shuttle cations between subcellular compartments, including from the cytosol into secretory vesicles or chloroplasts (reviewed by Hanikenne and Baurain, 2014). Only HMA4 has appeared in plasmodesmal proteomes to date (Fernandez-Calvino *et al*., 2011), and this localization has yet to be confirmed, suggesting that P_1B_-type ATPases are unlikely to be core regulators of plasmodesmal function.

Beyond Arabidopsis, catalogues of *HIPP* and *HPP* genes have been assembled from numerous plant species, including lycophytes, and monocotyledonous and dicotyledonous flowering plants (Table 1). Tandem and segmental duplications have contributed to the major expansion of select families (Khan *et al*., 2019; Li *et al*., 2020; Ye *et al*., 2022), causing some species to accumulate >50 *HIPP* genes. Notably, HIPPs appear to be exclusive to tracheophytes. Thus, HIPPs, like PDLPs (Lee, 2014), seem to have arisen after the division of vascular and non-vascular plants. As plasmodesmata are present in both lineages, this suggests that HIPPs may be regulators of plasmodesmal function, rather than essential structural components or governors of plasmodesmal morphogenesis.

## A protein perspective: HMA domains are frequently targeted by pathogens

### HMA domains as effector host targets

While there remains much to learn about HMA proteins in the context of plasmodesmata, evidence is continually emerging for their involvement in plant–pathogen interactions. HPP/HIPPs from diverse plant species have been identified as direct targets of pathogen-derived molecules, including secreted effector proteins and a viral movement protein (Table 2).

**Table 2.** HMA proteins implicated in plant–pathogen interactions*^a^*

Host	HMA protein	#HMA	M/LXCXXC region	Pathogen	Pathogen protein	SF	Reference
Arabidopsis	AtCCS AT1G12520	1	MTCEGC	*Golovinomyces orontii*	OEC45	—	Weβling *et al*. (2014)
Arabidopsis	AtHIPP13 AT5G52750	1	−*^b^*	*Hyaloperonospora arabidopsidis*	ATR13	—	Mukhtar *et al*. (2011)
Arabidopsis	AtHIPP19 AT3G21490	1	MHCNDC	*Hyaloperonospora arabidopsidis*	HaRxL44	—	Mukhtar *et al*. (2011)
				*Golovinomyces orontii*	OEC56 OEC115	—	Weβling *et al*. (2014)
Arabidopsis	AtHIPP26 AT4G38580	1	MDCEGC	*Xanthomonas campestris*	XopK	—	González-Fuente *et al*. (2020)
Arabidopsis	AtHIPP27 AT5G66110	1	MDCEGC	*Heterodera schachtii*	—	Y	Radakovic *et al*. (2018)
				*Meloidogyne incognita*	—	Y	Dutta *et al*. (2023)
Arabidopsis	AtHMP35 AT4G16380	1	LDCAKC	*Hyaloperonospora arabidopsidis*	HaRxL44	—	Mukhtar *et al*. (2011)
Arabidopsis	CCP (AtHPP05) AT4G05030	1	MRCDKC	*Pseudomonas syringae* pv. *tomato* DC3000	—	N	Chai *et al*. (2020)
Arabidopsis	AthMAD1 (AtHMP09) AT1G51090	1	LNCSKC	*Pseudomonas syringae* pv. *tomato* DC3000	—	Y	Imran *et al*. (2016)
*Citrus sinensis*	CsHIPP03 Cs_ont_2g000480	2	MHCEAC MHCEAC	Candidatus Liberibacter asiaticus	SDE34	−*^c^*	Hu *et al*. (2023)
*Eragrostis curvula*	EcHIPP43 EJB05_15595	1	MDCEGC	*Magnaporthe oryzae*	Pwl2	—	Were *et al*. (2025)
*Haynaldia villosa*	HIPP1-V KM216991	1	MDCEGC	*Blumeria graminis* f. sp. *tritici*	—	N*^d^*	Wang *et al*. (2023)
				*Magnaporthe oryzae*	Pwl2	—	Were *et al*. (2025)
*Hordeum vulgare*	HvHIPP43 HORVU.MOREX. r3.2HG0164110.1 or HORVU.MOREX.r3.3HG0267040.1*^e^*	1	MDCEGC	*Magnaporthe oryzae*	Pwl1 Pwl2 PWL2–3 Pwl3 Pwl4	—	Were *et al*. (2025)
*Nicotiana benthamiana*	NbHIPP26 Niben101Scf 07109g05004	1	MDCEGC	Potato mop-top virus	TGB1	Y	Cowan *et al*. (2018)
*Oryza sativa*	OsHIPP05 LOC_Os04g32850	1	LQCCRC	*Magnaporthe oryzae*	—	Y	Fukuoka *et al*. (2009); Nakao *et al*. (2011)
*Oryza sativa*	OsHIPP19 LOC_Os04g39350	1	MPCEKS	*Magnaporthe oryzae*	AVR-PikA AVR-PikC AVR-PikD AVR-PikE AVR-PikF	N	Maidment *et al*. (2021); Oikawa *et al*. (2024)
*Oryza sativa*	OsHIPP20 LOC_Os04g39010	1	MPCEKS	*Magnaporthe oryzae*	AVR-PikD	Y	Oikawa *et al*. (2024)
*Oryza sativa*	OsHIPP28 LOC_Os10g14870	2	MHCEGC MHCDAC	*Rhizoctonia solani*	RsMf8HN	—	Niu *et al*. (2023)
*Oryza sativa*	OsHIPP43 LOC_Os01g32330	1	MDCEGC	*Magnaporthe oryzae*	Pwl1 Pwl2 Pwl2-2 Pwl2-3 Pwl3 Pwl4	Y	Zdrzałek *et al*. (2024)
*Oryza sativa*	OsHPP03 LOC_Os02g37290	1	MASDKC	*Magnaporthe oryzae*	AVR-PikD	—	Oikawa *et al*. (2024)
*Oryza sativa*	OsHPP04 LOC_Os02g37300	1	MSCDKC	*Magnaporthe oryzae*	AVR-PikD	N	Oikawa *et al*. (2024)
				*Meloidogyne graminicola*	MgMO298	Y	Song *et al*. (2021); Huang *et al*. (2023)
*Setaria italica*	sHMA94 SETIT_012363mg	1	MPSPKN	*Magnaporthe oryzae*	AVR-PikD APikL2F	—	Bentham *et al*. (2021) *^f^*
*Setaria italica*	sHMA25 SETIT_011333mg	1	MSNAKS	*Magnaporthe oryzae*	AVR-PikD APikL2F APikLA	—	Bentham *et al*. (2021) *^f^*
*Setaria italica*	SitiHIPP43 SETIT_003232mg	1	MDCEGC	*Magnaporthe oryzae*	Pwl2	—	Were *et al*. (2025)
*Triticum aestivum*	TaHIPP1 TraesCS2A 01G064900	1	IDCEGC	*Puccinia striiformis* f. sp*. tritici*	—	Y	Zhang *et al*. (2015)
*Triticum aestivum*	TaesHIPP43 TraesCS3D 02G209500	1	MDCEGC	*Magnaporthe oryzae*	Pwl2	—	Were *et al*. (2025)

^*a*^For each entry, gene identifiers and known pathogen-derived interactors are listed, as are the number of HMA/HMA-like domains (#HMA) and the sequence equivalent to the metal-binding motif (M/LXCXXC) in their β1–α1 loop region(s). Whether or not the HMA protein has been identified as a susceptibility factor (SF) in the pathosystem of interest is indicated by Y (yes), N (no), or — (not tested).

^*b*^The AtHIPP13 sequence included no conserved residues of the M/LXCXXC motif in the β1–α1 region.

^*c*^Although not investigated in *C. sinensis*, the function of CsHIPP03 as a susceptibility factor was proposed, as independent silencing of three homologous transcripts (*NbHIPP3.1*, *NbHIPP3.2*, and *NbHIPP3.3*) enhanced *N. benthamiana* resistance to *Pto* DC3000 *ΔhopQ1-1*.

^*d*^Resistance to *Bgt* was assessed in *T. aestivum HIPP1-V* overexpression lines.

^*e*^In a yeast two-hybrid assay, Pwl2 bound two HvHIPP43 paralogues, both of which possess an M/LXCXXC region reading MDCEGC.

^*f*^A total of 56, 19, and nine candidate sHMA-binding partners were identified for AVR-PikD, APikL2A, and APikL2F, respectively, originating from *O. sativa*, *S. italica*, and *T. aestivum*. Only the two structurally characterized effector–HMA pairs from this study are included here.

**Fig. 2.**
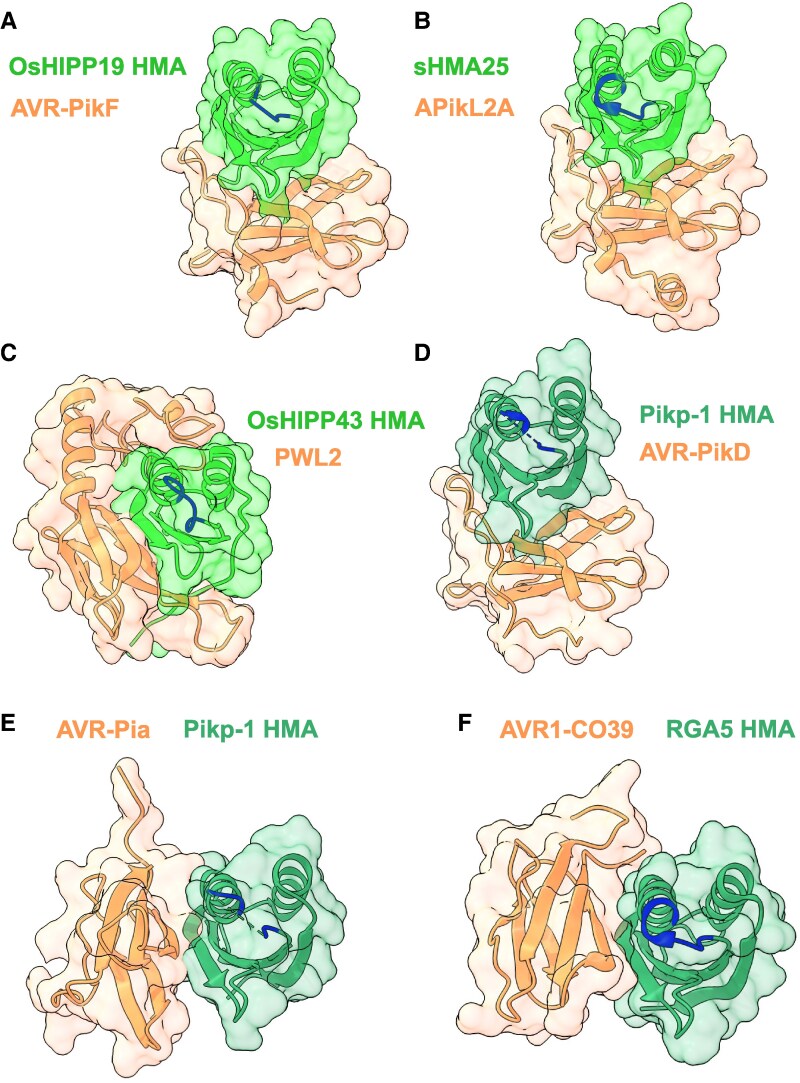
Structurally characterized *M. oryzae* effectors in complex with the HMA or HMA-like domains of host target proteins and ID-NLRs. Crystal structures of (A) AVR-PikF with the HMA-like domain of OsHIPP19 from rice (7B1I) (Maidment *et al*., 2021); (B) APikL2A with the HMA-like domain of sHMA25 from foxtail millet (7NLJ) (Bentham *et al*., 2021); (C) Pwl2 with the HMA domain of OsHIPP43 from rice (8R7D) (Zdrzałek *et al*., 2024); (D) AVR-PikD with the HMA-like ID of Pikp-1 from rice (5A6W) (Maqbool *et al*., 2015); (E) AVR-Pia with the HMA-like ID of Pikp-1 (6Q76) (Varden *et al*., 2019); (F) AVR1-CO39 with the HMA-like ID of RGA5 from rice (5ZNG) (Guo *et al*., 2018). Complexes are displayed such that the HMA/HMA-like domains are in equivalent orientations. Domains from HPP/HIPP host targets are coloured light green. Domains from ID-NLRs are coloured dark green. Regions of ribbon models equivalent to the metal-binding motif (M/LXCXXC) in the β1–α1 loop region of HMA/HMA-like domains are coloured blue. Effectors are coloured orange.

The most well-studied examples of effector–HMA interactions originate from *Magnaporthe oryzae* (rice/wheat blast) pathosystems. OsHIPP19 is targeted by multiple AVR-Pik allelic effector variants (Maidment *et al*., 2021), and OsHIPP20 by the AVR-PikD variant (Oikawa *et al*., 2024). *In vitro* work revealed that OsHIPP43 is tightly bound by Pwl effectors (Zdrzałek *et al*., 2024), and HvHIPP43, a barley orthologue of OsHIPP43, was identified as a Pwl2 target using immunoprecipitation-MS (IP-MS) (Were *et al*., 2025). Furthermore, yeast two-hybrid screening efforts uncovered a plethora of small HMA (sHMA) proteins from rice, foxtail millet (*Setaria italica*), and wheat (*Triticum aestivum*) as potential targets of AVR-Pik and AVR-Pik like (APikL) effectors (Bentham *et al*., 2021). Importantly, some *M. oryzae* effectors listed above are known to directly bind the HMA/HMA-like domains of their respective targets (Fig. 2A–C).

In addition to *M. oryzae*, targeting of host HMA proteins takes place across phytopathogen kingdoms. OsHPP04 is a target of the root knot nematode (*Meloidogyne graminicola*) effector MgMO298 (Song *et al*., 2021), and was also identified as a candidate binding partner of AVR-PikD (Oikawa *et al*., 2024). Meanwhile, NbHIPP26 from *N. benthamiana* was identified as a target of the potato mop-top virus (PMTV) movement protein TGB1, although in this case, the isoprenylation motif of NbHIPP26 was found to be critical for TGB1 association, rather than the HMA domain (Cowan *et al*., 2018). In Arabidopsis studies, extensive yeast two-hybrid screens identified candidate HMA domain-containing targets of effectors from the powdery mildew fungus, *Golovinomyces orontii*, downy mildew oomycete, *Hyaloperonospora arabidopsidis*, and the bacterium, *Xanthomonas campestris* (Table 2). Considered together, the repeated appearance of HPP and HIPP proteins in non-biased searches for host targets across diverse pathosystems hints at widespread roles for HMA proteins in plant immunity.

### HMA domains as bait for effectors

HMA-like domains have been identified within plant resistance proteins, including nucleotide-binding leucine-rich repeat (NLR) receptors, and tandem kinase proteins (TKPs), which form part of the intracellular defence signalling system of plants. Typically, NLRs are composed of a C-terminal leucine-rich repeat (LRR) domain, central NB-ARC (nucleotide-binding adaptor shared by APAF-1, R proteins, and CED-4) domain, and a variable N-terminal signalling domain, capable of triggering localized cell death when activated, for example via direct or indirect recognition of effectors (for a recent review, see Locci and Parker, 2024). Additionally, some NLRs contain integrated domains (IDs), including WRKY, WD-40, and, of relevance here, HMA-like domains (Sarris *et al*., 2016; Grund *et al*., 2019; Table 3). It is hypothesized that NLR IDs structurally mimic host targets of their cognate effectors, serving as bait to enable robust effector-triggered responses (Cesari *et al*., 2013, 2014; Maqbool *et al*., 2015; Ortiz *et al*., 2017; Mukhi *et al*., 2021). Beyond NLRs, TKPs are an emerging class of resistance proteins that commonly contain non-kinase IDs (Reveguk *et al*., 2025; Table 3), hinting at another context in which plants might exploit the HMA structure for effector recognition.

**Table 3.** Examples of plant resistance proteins with HMA-like domains and their corresponding helper NLRs and effectors, where known^a^

Plant	Sensor	Domain architecture	M/LXCXXC region	Helper	Pathogen	Effector(s)	References
*Hordeum vulgare*	RPG1	HMA-Pseudokinase-kinase	−*^a^*	Unknown	*Puccinia graminis f. sp. tritici*	Unknown	Brueggeman *et al*. (2002); Nirmala *et al*. (2006); Reveguk *et al*. (2025)
*Hordeum vulgare*	MLA3	CC-NB-LRR	−*^b^*	—	*Magnaporthe oryzae*	Pwl2	Gómez De la Cruz *et al*. (2024, Preprint)
*Oryza sativa*	Pikm-1	CC-HMA-NB-LRR	MVDDKS	Pikm-2	*Magnaporthe oryzae*	AVR-PikA AVR-PikD AVR-PikE AVR-Mgk1	De la Concepcion *et al*. (2018); Sugihara *et al*. (2023)
*Oryza sativa*	Pikp-1	CC-HMA-NB-LRR	MEGNNC	Pikp-2	*Magnaporthe oryzae*	AVR-PikD AVR-Mgk1	De la Concepcion *et al*. (2018); Sugihara *et al*. (2023)
*Oryza sativa*	RGA5	CC-NB-LRR-HMA	MPCGKS	RGA4	*Magnaporthe oryzae*	AVR-Pia AVR1-CO39	Cesari *et al*. (2013); Ortiz *et al*. (2017)

The sequence equivalent to the metal-binding motif (M/LXCXXC) in the β1–α1 loop region of the HMA-like domain is given. CC, coiled coil; NB, nucleotide-binding; LRR, leucine-rich repeat.

^*a*^The RPG1 sequence included no conserved residues of the M/LXCXXC motif in the β1–α1 loop.

^*b*^Although MLA3 does not possess a full HMA-like fold, the final three repeats of the LRR structurally mimic the Pwl2-binding surface of the OsHIPP43 HMA domain. This region does not include a β1–α1 loop.

Among ID-NLRs, the recognition profiles of Pik-1 allelic variants from rice have been studied in depth, and effector binding specificity is known to be determined by their polymorphic HMA-like IDs (Maqbool *et al*., 2015; De la Concepcion *et al*., 2018, 2019, 2021). Structural studies suggest that HMA binding might have evolved convergently among *M. oryzae* effectors, as some form distinct interfaces with their respective targets, despite sharing conserved folds (Fig. 2). Overall, the repeated integration of HMA-like structures into plant resistance proteins corroborates the hypothesis that HPPs and HIPPs are frequently targeted by pathogens as a result of important, but poorly understood, roles in plant immunity.

## A genetic perspective: HMA proteins contribute to plant disease outcomes

RNA-sequencing and microarray studies have revealed a range of expression profiles for *HPP/HIPP* genes under various abiotic and biotic stresses, suggesting a degree of functional divergence within the families (de Abreu-Neto *et al*., 2013). However, targeted quantitative reverse transcription–PCR (RT–qPCR) experiments frequently highlight genes encoding pathogen-targeted HPP/HIPPs as being up-regulated in an infection context: *NbHIPP26* in response to PMTV (Cowan *et al*., 2018); *OsHIPP43* in response to *M. oryzae* (Were *et al*., 2025); and *OsHPP04* in response to *Meloidogyne graminicola* (Song *et al*., 2021), among others whose products are not yet characterized as pathogen host targets (Zhang *et al*., 2015; Zschiesche *et al*., 2015; Radakovic *et al*., 2018; Chai *et al*., 2020; Dutta *et al*., 2023; Wang *et al*., 2023). To investigate the potential consequences of these expression changes within a given pathosystem, genetic studies exploiting mutants and overexpression lines have been key. To date, both negative and positive regulators of defence have been identified amongst HMA proteins, making it especially challenging to assign a consensus immune function.

### HMA proteins as susceptibility factors

Multiple HIPP and HPP genes have been labelled as host susceptibility factors based on the observation that their loss of function results in enhanced resistance to a particular pathogen (Table 2). For example, the recessive *pi21* locus in rice encodes a loss-of-function *OsHIPP05* mutation that enhanced resistance to 10 races of *M. oryzae* (Fukuoka *et al*., 2009). Remarkably, heterologous expression of *OsHIPP05* in Arabidopsis (a non-host) enabled growth of *M. oryzae* hyphae, supporting the possibility that the encoded protein functions as a negative regulator of host basal resistance (Nakao *et al*., 2011).

While Oikawa *et al*. (2024) showed that OsHIPP19 and OsHIPP20 were each stabilized in complex with the effector AVR-PikD, deletion of *OsHIPP19* did not significantly impact rice susceptibility to *M. oryzae*. However, *OsHIPP20* deletion enhanced resistance, suggesting that AVR-PikD binding of this host target might ordinarily favour blast disease progression (Oikawa *et al*., 2024). HvHIPP43 was also implicated as a susceptibility factor, as barley plants overexpressing this HIPP were more susceptible to *M. oryzae* (Were *et al*., 2025).

OsHPP04 might likewise function as a susceptibility factor but in a pathogen-specific manner. Although a knockout mutant of *OsHPP04* in rice showed unaltered resistance to *M. oryzae* (Oikawa *et al*., 2024), an alternative mutant allele of the same gene generated by Huang *et al*. (2023) supported significantly fewer root knot nematodes (*M. graminicola*) than wild-type rice. In Arabidopsis, *AtHIPP27* function promoted susceptibility to two different nematode species: *Heterodera schachtii* (Radakovic *et al*., 2018) and *Meloidogyne incognita* (Dutta *et al*., 2023), while *AtHMAD1* (*AtHMP09*) promoted susceptibility to *Pseudomonas syringae* pv. *tomato* (*Pto*) DC3000 (Imran *et al*., 2016). Whether or not the HMA domains of AtHIPP27 and AtHMAD1 are directly targeted by effectors remains to be determined.

Products of susceptibility genes can promote pathogen establishment via different mechanisms, including facilitation of host recognition and entry, pathogen nutrient acquisition and movement, or via suppression of host immune responses (Garcia-Ruiz *et al*., 2021). Although not the case for all susceptibility factors described above (e.g. AtHIPP27, Radakovic *et al*., 2018), genetic evidence suggests that some HMA proteins may negatively regulate pathogen elicitor-induced reactive oxygen species (ROS) bursts and/or defence gene expression during infection (Fukuoka *et al*., 2009; Huang *et al*., 2023; Were *et al*., 2025). Evidence for HPP/HIPP-mediated negative regulation of basal plant immune responses is thus building; however, the intracellular mechanisms underlying this suppression are unclear.

### HMA proteins as positive regulators of defence

In contrast to the susceptibility factors described above, a select few HMA proteins have been shown to promote host resistance to pathogens. HIPP1-V is a single HMA domain-containing HIPP from the diploid wheat relative, *Haynaldia villosa*, whose heterologous overexpression in hexaploid wheat resulted in enhanced resistance to the powdery mildew fungus, *Blumeria graminis* f. sp. *tritici* (*Bgt*) (Wang *et al*., 2023). To investigate the underpinning mechanism, Wang *et al*. (2023) identified CMPG1-V as a HIPP1-V binding partner using a yeast two-hybrid approach. *CMPG1-V* was previously characterized as a resistance gene (Zhu *et al*., 2015), suggesting that HIPP1-V and CMPG1-V might co-operate to promote disease resistance. Specifically, HIPP1-V is proposed to recruit CMPG1-V to the plasma membrane following its isoprenylation, thereby initiating defence signalling (Wang *et al*., 2023).

Another positive regulator of plant immunity is AtHPP05, also named CCP (copper chaperone induced by pathogens) (Chai *et al*., 2020). *CCP* overexpression enhanced Arabidopsis resistance to *Pto* DC3000, while its deletion enhanced susceptibility. Although heavy metal binding was not explicitly demonstrated, CCP is homologous to human ATOX1, and is proposed to bind Cu via its HMA domain, facilitating its homodimerization and nuclear import. There, CCP was found to bind the transcription factor TGA2, activating transcription of the defence gene *PR1* (Chai *et al*., 2020), which encodes a widely studied antimicrobial signalling protein (Han *et al*., 2023).

Considering the examples above, whether HMA proteins are positive or negative regulators of immunity does not seem to correlate with their number of HMA domains (Table 2), pathogen-induced changes in expression patterns, or subcellular localization. Instead, the influence of HPP/HIPPs on disease outcomes may depend on (i) if/how the protein is targeted by effectors (which so far remains to be determined for HIPP1-V and CCP), (ii) the consequences of this (for instance, stabilization versus degradation of the HMA protein), and (iii) the distinct protein–protein interaction networks of HPP/HIPP family members. To better understand the variety of immune-related functions reported for HMA proteins, more data surrounding these three aspects are required.

## Lessons from microbes and beyond

As HMA proteins have emerged as host targets of diverse pathogen-derived proteins and/or determinants of host susceptibility, we draw on observations made in plant pathology studies, as well as those focused on plant development and abiotic stress tolerance, to generate hypotheses about their intracellular functions, including at plasmodesmata.

### HPP/HIPPs regulate phytohormone signalling

HPP and HIPP proteins have been implicated in phytohormone regulation, including in developmental and defensive contexts. Firstly, a handful of ER-associated Arabidopsis HIPPs were characterized as regulators of cytokinin signalling by Guo *et al*. (2021). Overexpression of *AtHIPP01* led to enhanced degradation of the cytokinin-degrading enzyme CKX1 (cytokinin oxidase/dehydrogenase), increasing cytokinin sensitivity and severely stunting rosette growth. In this work, both AtHIPP01 and AtHIPP07 were observed at plasmodesmata, yet the functional relevance of this was not discussed. Exogenous cytokinin application was recently shown to enhance cell-to-cell movement of fluorescent proteins in *N. benthamiana* leaves (Horner and Brunkard, 2021), speculatively via rapid *de novo* formation of secondary (post-cytokinesis) plasmodesmal pores—a phenomenon previously demonstrated in the shoot apical meristem of *Sinapis alba* (Ormenese *et al*., 2006). Whether or not HIPP-mediated modulation of cytokinin responses ordinarily governs secondary plasmodesmata formation remains to be studied.

Secondly, pathology studies hint at a role for HMA proteins in salicylic acid (SA) signalling. For example, AtHIPP03 was implicated as a negative regulator of SA responses, based on co-expression analysis of Arabidopsis plants constitutively expressing this HIPP (Zschiesche *et al*., 2015). AtHMAD1 is also thought to negatively regulate SA signalling, as *athmad1* mutants displayed increased expression of systemic acquired resistance (SAR) marker genes post-inoculation with *Pto* DC3000 (Imran *et al*., 2016). In contrast, transcripts related to SA signalling, as well as accumulation of SA itself, were increased in wheat plants overexpressing *HIPP1-V* (Wang *et al*., 2023), suggesting that this HIPP might positively regulate SA-responsive pathways. Likewise, CCP may promote SA responses via its interaction with TGA2, a known regulator of SA-dependent gene expression (Boyle *et al*., 2009; Chai *et al*., 2020). As SA is an established elicitor of plasmodesmal closure via callose deposition (Wang *et al*., 2013; Huang *et al*., 2019; Tee *et al*., 2023), it is tempting to speculate that HMA proteins could regulate plasmodesmal aperture indirectly via SA. However, why these proteins should require plasmodesmal localization to do so remains unclear.

### HPP/HIPPs regulate oxidative stress tolerance

ROS are established second messengers in plant signalling, and their accumulation is associated with both abiotic and biotic stress (for reviews, see Castro *et al*., 2021; Mittler *et al*., 2022). On perception of stressful stimuli, ROS build-up can occur in chloroplasts and mitochondria as a consequence of electron transport chain malfunction. ROS may also be actively produced via oxidases in peroxisomes, and in the apoplast via cell wall peroxidases and respiratory burst oxidase homologue (RBOH) enzymes. To prevent excessive oxidative damage to the cellular machinery during stress, plants utilize ROS-scavenging and transport proteins, as well as non-enzymatic antioxidants. To explain their apparent involvement in so many plant processes and pathosystems (de Abreu-Neto *et al*., 2013; Table 2), could HMA proteins somehow regulate these broad-spectrum stress signals?

The prototypical HMA protein ATX1 was first identified in a genetic screen for antioxidant factors capable of functionally substituting for *S. cerevisiae* Cu/Zn-SOD 1 (SOD1) (Lin and Culotta, 1995), a metalloenzyme that catalyses the dismutation of damaging superoxide, forming hydrogen peroxide and oxygen (Bermingham-McDonogh *et al*., 1988). Although not responsible for direct delivery of metal cofactors to SOD1 (Culotta *et al*., 1997), ATX1 is thought to potentially dampen oxidative stress using its own (albeit inefficient compared with SOD1) superoxide dismutase activity (Portnoy *et al*., 1999).

In plants, participation in ROS scavenging has similarly been suggested for HPPs. Through the use of alternative translation start sites, *AtCCS* transcripts were found to encode both cytosolic- and chloroplast-targeted protein isoforms, enabling activation of three Arabidopsis Cu/Zn-SOD (CSD) enzymes: CSD1 (cytoplasmic), CSD2 (stromal), and CSD3 (peroxisomal) (Abdel-Ghany *et al*., 2005; Chu *et al*., 2005). Later, a study in the *M. graminicola*–rice system suggested that OsHPP04 may deliver Cu cofactors to cytosolic Cu/Zn-SOD 2 (cCu/Zn-SOD2) and, upon nematode infection, binding of the MgMO298 effector to OsHPP04 is thought to promote ROS scavenging, thereby dampening host immune responses (Song *et al*., 2021). Besides nematodes, it is conceivable that other phytopathogens might have evolved to exploit HPP-mediated delivery of metal ion cofactors to enhance enzymatic ROS scavenging.

Recent work by Were *et al*. (2025) uncovered a potential link between a plasmodesmata-localized HIPP and ROS homeostasis, as barley plants overexpressing *HvHIPP43* exhibited reduced ROS bursts in response to pathogen elicitors flg22 and chitin. It is not yet established whether these plants scavenged ROS more efficiently, or were compromised in ROS production. Nevertheless, it is proposed that binding of the *M. oryzae* effector Pwl2 might somehow enhance the ability of HvHIPP43 to negatively regulate ROS signalling, as similar phenotypes were observed in *HvHIPP43*- and *Pwl2*-overexpressing plants (Were *et al*., 2025). Currently, it is unclear how these phenotypes relate to the plasmodesmal localization of HvHIPP43, which live cell imaging suggests to be compromised by Pwl2 (Were *et al*., 2025).

In contrast, HIPP1-V was suggested to promote ROS accumulation, as levels of oxidative response-related transcripts, NADPH oxidase enzymes, and hydrogen peroxide were increased in wheat plants overexpressing this HIPP, which may have contributed to their increased *Bgt* resistance (Wang *et al*., 2023). Unlike the mechanisms described for AtCCS and OsHPP04 above, exactly how HvHIPP43 and HIPP1-V might modulate ROS signalling remains unknown.

As an alternative to metallochaperone-based models, one could hypothesize that HMA domains regulate oxidative stress tolerance by participating in redox reactions. ROS accumulation generates local oxidative environments within cellular compartments, and is thus tightly linked to redox homeostasis. To combat oxidative stress, plants maintain large pools of antioxidants, including low molecular weight compounds such as glutathione, a tripeptide of Glu–Cys–Gly, which possess a solvent-accessible thiol (-SH) group (Jozefczak *et al*., 2012).

With exposed thiol groups as part of their CXXC metal-binding motif(s), it is possible that HPPs and HIPPs might also fluctuate between oxidized and reduced forms. Indeed, ATOX1 from humans is oxidized by glutathione, resulting in intramolecular disulfide bond formation that blocks Cu co-ordination in the HMA domain (Hatori *et al*., 2012; Brose *et al*., 2014). As redox environments are predicted to vary between cell types and subcellular compartments, it is proposed that ATOX1 couples Cu distribution with cellular redox status to regulate mammalian cell viability and differentiation (reviewed in depth by Hatori and Lutsenko, 2016). Whether or not HMA proteins function as key redox regulators in plants, as well as the consequences of this for ROS detoxification, remains to be studied.

In summary, multiple lines of evidence point towards roles for HMA proteins in ROS homeostasis, which has the potential to underpin their involvement in both abiotic and biotic stress tolerance. However, much of this evidence comes from the study of HPPs or mammalian HMA domains, leaving the question of how HIPPs alter plant ROS metabolism unanswered.

### HPP/HIPPs might be translocated intracellularly to regulate transcription

Live-cell imaging has uncovered HMA proteins in multiple subcellular compartments, from the cytoplasm and nucleus, to the plasma membrane and plasmodesmata (Fukuoka *et al*., 2009; Zhang *et al*., 2015; Cowan *et al*., 2018; Chai *et al*., 2020; Song *et al*., 2021; Wang *et al*., 2023; Huang *et al*., 2024; Oikawa *et al*., 2024; Were *et al*., 2025). However, the motifs and/or structural features that promote and maintain these localizations remain largely unknown. Nevertheless, studies on the *N. benthamiana* and Arabidopsis HIPP26 homologues have shed light on this.

Visualized with an N-terminal fluorescent tag, NbHIPP26 was found to localize to *N. benthamiana* nuclei (including the nucleoplasm and nucleoli) and to the plasma membrane, with enrichment at plasmodesmata (Cowan *et al*., 2018). However, when co-expressed with the PMTV movement protein TGB1 in a bimolecular fluorescence complementation (BiFC) experiment, NbHIPP26 predominantly localized to nucleoli and microtubules. These observations prompted Cowan *et al*. (2018) to propose a model whereby TGB1 binds and shuttles NbHIPP26 to the nucleus of vascular parenchyma cells (where *NbHIPP26* promoter activity is concentrated), promoting drought tolerance and long-distance PMTV movement via an unknown mechanism. *AtHIPP26* promoter activity was similarly enriched in Arabidopsis vascular tissue, and the AtHIPP26 protein localized to the nuclei of onion (*Allium cepa*) epidermal cells (Barth *et al*., 2009). In nuclei, AtHIPP26 is thought to manipulate drought-responsive gene expression via its binding partner, zinc finger homeobox protein ATHB29 (Barth *et al*., 2009). It is conceivable that the TGB1–NbHIPP26 complex might also manipulate transcription factor function to reprogramme *N. benthamiana* vascular cells.

AtHIPP26 is not the only HMA protein to bind transcription factors. Indeed, several HIPPs were suggested to bind ATHB29 in a yeast two-hybrid experiment, namely AtHIPP20, 21, 23, 24, 27, and 30, with AtHIPP30 showing additional interactions with ATHB21 and ATHB30 (Barth *et al*., 2009). Also, a BiFC experiment in *N. benthamiana* suggested that CqHIPP34 from quinoa (*Chenopodium quinoa*) bound CqZF-HD14, another positive regulator of drought tolerance (Sun *et al*., 2022). As mentioned earlier, CCP binds the bZIP transcription factor TGA2, recruiting it to the promoter of defence gene *PR1* (Chai *et al*., 2020).

As multiple HMA proteins appear to operate in cell nuclei, this raises mechanistic questions. Live imaging suggests that the TGB1–NbHIPP26 complex could be transported from plasmodesmata to nuclei via microtubules (Cowan *et al*., 2018). In this case, a combination of C-terminal isoprenylation and *S*-acetylation of a central Cys residue promoted membrane association of NbHIPP26, whereas mutation of the CXXC motif in the HMA domain had no noticeable effect on localization (Cowan *et al*., 2018). Consistent with this, mutation of the isoprenylation motif compromised plasmodesmal localization of other HIPPs: AtHIPP07 and HvHIPP43 (Guo *et al*., 2021; Were *et al*., 2025). Lipid modifications might also regulate the nuclear distribution of HIPPs. AtHIPP26 formed speckles within the nucleoplasm of onion cells, but when its isoprenylation motif was mutated, nuclear distribution was more homogenous (Barth *et al*., 2009). As isoprenylation has been documented to influence both protein–protein and protein–lipid interactions (Crowell, 2000; Wang *et al*., 2020), this phenomenon could speculatively be explained by facilitation or inhibition of AtHIPP26 binding to other nuclear proteins.

In contrast, CCP lacks an isoprenylation motif, and its nuclear import in Arabidopsis protoplasts required a functional CXXC motif and nuclear localization signal (KKVGF) near its C-terminus (Chai *et al*., 2020). According to Barth *et al*. (2009), AtHIPP26 likewise possesses a nuclear localization signal (DCSHGHKIKKRK), but this is found near the N-terminus of the protein. Thus, although it seems that multiple HMA proteins carry out signalling functions in nuclei, the routes they take to get there are not necessarily conserved.

### HPP/HIPPs might regulate plasmodesmal aperture

Some pathogen-targeted plasmodesmata-located HIPPs are known susceptibility factors (Cowan *et al*., 2018; Oikawa *et al*., 2024; Were *et al*., 2025), hinting that putative HIPP functions in immunity and plasmodesmal regulation could be linked. Plasmodesmata are critical during infection, as they offer routes for the intercellular spread of pathogens and their secreted effectors. Indeed, imaging-based screens have revealed cell-to-cell mobility to be common among nucleocytoplasmic effectors from the fungal pathogen *Colletotrichum higginsianum* (Ohtsu *et al*., 2024), the oomycete *H. arabidopsidis* (Liu *et al*., 2022, Preprint), and the bacterium *Pto* DC3000 (Li *et al*., 2021). Such mobile effectors could feasibly allow pathogens to suppress host defences ahead of their primary invasion front.

To enhance intercellular trafficking and overcome elicitor-induced closure of plasmodesmata, pathogen-mediated manipulation of plasmodesmal function is hypothesized to be widespread (Cheval and Faulkner, 2018). Viral movement proteins are known to target and open plasmodesmal channels in a variety of ways, including via alteration of callose metabolism, cytoskeleton dynamics, and tubule formation (for reviews, see Lucas, 2006; Taliansky *et al*., 2008; Reagan and Burch-Smith, 2020). Elsewhere, plasmodesmata-located effectors from bacteria, fungi, and oomycetes have been identified, some of which are modifiers of plasmodesmal function. For instance, HaRxL77 from *H. arabidopsidis* (Liu *et al*., 2022, Preprint), ChEC8, ChEC127, and ChEC132 from *C. higginsianum* (Ohtsu *et al*., 2024), and the co-operating effector pair, secreted in xylem 5 (Six5) and Avr2 (Six3) from *Fusarium oxysporum* (Cao *et al*., 2018) can enhance intercellular trafficking via unknown mechanisms. While Six5 does not modify plasmodesmal callose (Blekemolen *et al*., 2022), RxRL3 from the oomycete *Phytophthora brassicae* and HopO1 from *Pto* DC3000 both suppress callose accumulation to enhance plasmodesmal trafficking capacity (Aung *et al*., 2020; Tomczynska *et al*., 2020). Although empirical evidence is currently lacking, one could hypothesize that plasmodesmal HMA proteins might be endogenous regulators of plasmodesmal aperture, and are thus targeted by pathogens to maintain cell-to-cell connectivity during infection.

If HPPs and/or HIPPs do regulate plasmodesmal function, could ROS be involved? A handful of plant HMA proteins have been implicated in ROS homeostasis (discussed above) and, as part of elicitor-induced signalling cascades, ROS are known regulators of plasmodesmal dynamics. For example, extracellular flg22 or chitin perception culminates in RBOHD activation via independent pathways, triggering apoplastic ROS accumulation and closure of plasmodesmata via CALLOSE SYNTHASE 1 (CALS1)-mediated callose deposition (Xu *et al*., 2017; Cheval *et al*., 2020; Tee *et al*., 2023). With this in mind, it is puzzling to consider how intracellular HMA proteins, which lack transmembrane and extracellular domains, could contribute to plasmodesmal regulation via apoplastic ROS. A greater understanding of HPP/HIPP binding partners at plasmodesmata would help address this.

Alternatively, as some HMA proteins have been identified as positive regulators of defence, it is possible that a subset may reside at plasmodesmata as part of a stress perception system. Reminiscent of IDs in immune receptors (see ‘HMA domains as bait for effectors’), perhaps some defence-promoting HPP/HIPPs employ their HMA domains as bait, perturbing cell-to-cell movement and/or virulence functions of effectors, thus slowing infection spread. Building on the established role of plasmodesmata as signalling hubs within the plasma membrane, and the potential nuclear functions of HPPs and HIPPs discussed above, it is possible that effector–HMA binding events at plasmodesmata might trigger downstream immune responses, including transcriptional reprogramming to fortify host defences. Further characterization of the intracellular functions of defence-promoting HMA proteins is required to test these hypotheses.

## What microbes aren’t telling us: HPP/HIPP function in heavy metal tolerance

Perhaps one of the biggest challenges in HMA protein research is uncovering empirical links between three major themes discussed in this review: plasmodesmata, immunity, and heavy metal binding. Some of the first described HMA domains belonged to metallochaperones participating in ion transport within cells, including widely conserved Cu chaperones (see ‘A historical perspective: what makes a HMA domain?’). Unsurprisingly, in plants, HIPPs and HPPs have been implicated in regulating heavy metal tolerance, especially regarding the toxic non-nutrient metal, cadmium (Cd). AtHIPP06, 20, 21, 22, and 26 are induced by Cd exposure and positively regulate Cd tolerance in Arabidopsis (Suzuki *et al*., 2002; Gao *et al*., 2009; Tehseen *et al*., 2010). Following observations of higher Cd levels in overexpressing plants, AtHIPP44 was also implicated in regulating Cd uptake (Zhang *et al*., 2019). Meanwhile, 11 rice *HPP/HIPP* genes and three tea plant (*Camellia sinensis*) *HIPP* genes were up-regulated under Cd stress, and the growth of *oshipp42* rice mutants was marginally reduced under Cu, Zn, Cd, and manganese (Mn) stress (Khan *et al*., 2019; Wei *et al*., 2023). *OsHIPP29* and *HIPP1-V* overexpression enhanced Cd tolerance in rice and bread wheat, respectively (B.Q. Zhang *et al*., 2020; H. Zhang *et al*., 2020). Despite the growing number of studies reporting HPP/HIPP involvement in metal homeostasis, many questions regarding the underlying mechanisms remain.

Firstly, do all HPPs and HIPPs bind metal ions directly? Dykema *et al*. (1999) demonstrated that AtHIPP07 (originally ATFP3) bound Cu(II), Ni(II), or Zn(II) in a reversible manner within immobilized metal affinity chromatography columns. Subsequently, using different *in vitro* methods, AtHIPP26 (originally ATFP6) was discovered to bind Pb(II), Cd(II), and Cu(II) ions (Gao *et al*., 2009), AtHIPP03 was shown to bind Zn(II) (Zschiesche *et al*., 2015), and AtHIPP06 (originally CdI19 for Cd-induced 19) was shown to bind Cd(II), Cu(II), and Hg(II) (Suzuki *et al*., 2002). Despite this, evidence for *in planta* metal binding of HIPPs and HPPs is notably lacking, and the specificity and fate of associated metals are unclear. Also, one cannot rule out the possibility that HPP/HIPP regulation of heavy metal tolerance could be indirect, for example via regulation of ROS (see earlier), given that ROS production is induced by metal stress (Shahid *et al*., 2014). Alternatively, Zhou *et al*. (2023) proposed a transcription-based tolerance mechanism, as overexpression of *ApHIPP26* from *Arabis paniculata* enhanced Arabidopsis growth under Cd stress, accompanied by increased expression of genes involved in metal uptake and translocation.

Secondly, what can the number of HMA domains tell us about putative metallochaperone function? So far, the biological significance of a HPP or HIPP possessing one versus two HMA domains has not been directly studied. Structural characterization of HMA domains from yeast, bacteria, and humans suggests that a single HMA fold, complete with a CXXC motif, is sufficient for metal binding (Steele and Opella, 1997; Gitschier *et al*., 1998; Hung *et al*., 1998; Rosenzweig *et al*., 1999); however, metals have yet to appear in structures of plant HMA or HMA-like domains (Maqbool *et al*., 2015; De la Concepcion *et al*., 2018, 2021; Guo *et al*., 2018; Varden *et al*., 2019; Bentham *et al*., 2021; Maidment *et al*., 2021; Zdrzałek *et al*., 2024, Fig. 2), and the structures of double-HMA HPP/HIPP proteins have not been characterized.

It is plausible that a double-HMA architecture might increase the versatility of a given protein by facilitating association with more/different metal species. Interestingly, ATX1 from the cyanobacterium *Synechocystis* PCC6803 has been crystallized as a dimer, loaded with two or four Cu(I) ions (Badarau *et al*., 2010). The human and yeast ATX1 orthologues also dimerize in the presence of Cu (Tanchou *et al*., 2004; Miras *et al*., 2008), and X-ray crystallography revealed a distorted tetrahedral geometry involving four Cys residues at the Cu(I)-binding site of these dimers, stabilized by hydrogen bonds (Wernimont *et al*., 2000; Lee *et al*., 2017). Speculatively, depending on the relative orientation of their domains, a double-HMA protein might also be capable of binding Cu(I) or divalent cations such as Zn(II) in a tetrahedral environment, akin to the ‘structural’ metal-binding sites of some Zn finger proteins (Permyakov, 2021). Indeed, crystal structures suggest that ATOX1 from humans can bind Zn(II), Cd(II), and platinum [Pt(II)] as a homodimer (Wernimont *et al*., 2000; Belviso *et al*., 2016; Mangini *et al*., 2022). Such tetrahedral co-ordination sites might be better suited for metal sequestration and/or enhancing protein stability, as opposed to metal trafficking. Hinting at the possibility of metal-dependent HIPP–HIPP interactions, Guo *et al*. (2021) observed homodimerization of AtHIPP7 in yeast, which was abolished by Cys to Gly mutation in the CXXC motifs. Clearly, there remains much to learn about the chemistry and specificity of metal-binding sites in plant HMA domains.

Thirdly, why might proteins involved in metal homeostasis be localized to plasmodesmata? A possible clue comes from a study by O’Lexy *et al*. (2018) (also summarized by Barr *et al*., 2023) in which heavy metal species were reported to have distinct effects on plasmodesmal function in roots. Specifically, iron (Fe) stress reduced channel permeability in a CALS12-dependent manner, while Cu stress enhanced permeability in a β-1,3-glucanase-dependent manner. While the components involved in metal sensing and signalling in this context remain unknown, it is tempting to speculate that HIPPs could be involved; ‘capturing’ heavy metals and perhaps transferring them to metalloenzymes, thereby triggering signalling cascades that feed back to modify plasmodesmal function.

Finally, is metal binding relevant for HMA protein function in plant immunity? While the majority of host HMA proteins implicated in plant–microbe interactions sport a conserved metal-binding motif, there are notable exceptions: OsHIPP19, OsHIPP20, and OsHPP03 from rice, and sHMA94 and sHMA25 from foxtail millet (Table 2). Also, regions corresponding to the ‘metal-binding loop’ (between β1 and α1) of HMA-like IDs within rice NLRs Pik-1 and RGA5 do not include two Cys residues (Table 3), suggesting that metal binding is not required for baiting effectors. Consistent with this, in effector–HMA crystal structures, the β1–α1 loop rarely constitutes part of the binding interface (Fig. 2). Nevertheless, indirect evidence from a handful of studies suggests functional links between HPP/HIPP metallochaperone functions and plant immunity (Zhang *et al*., 2015; Chai *et al*., 2020; Song *et al*., 2021).

A speculative hypothesis is that heavy metals carried by HMA domains could be released during stress and employed as anti-microbial weapons, or delivered to intracellular defensive enzymes such as SODs and metallopeptidases (Moreau *et al*., 2013). Curiously, NONEXPRESSOR OF PATHOGENESIS-RELATED GENES 1 (NPR1), a transcriptional co-regulator of SA-responsive gene expression, was shown to bind SA via a co-ordinated transition metal ion (probably Cu, according to *in vitro* analyses) (Wu *et al*., 2012). Should HMA proteins be implicated in provision or removal of NPR1-associated metals, this would corroborate observations of HPP/HIPP influence on SA signalling.

Alternatively, evidence from studies of hyperaccumulator plants suggests that high concentrations of heavy metals might deter or kill herbivores and pathogens directly, referred to as the ‘elemental defence hypothesis’ (reviewed by Hörger *et al*., 2013; De *et al*., 2025). For example, hyperaccumulation of Zn, nickel (Ni), or Cd by *Thlaspi caerulescens* enhanced resistance to the apoplastic bacterium *P. syringae* pv. *maculicola* (Fones *et al*., 2010). Also, local accumulation of Zn around invasion sites was associated with increased resistance of Arabidopsis (a non-accumulator plant) to the fungal necrotroph *Plectosphaerella cucumerina* BMM (Escudero *et al*., 2022). Although evidence for elemental defence against intracellular pathogens is limited, one could imagine that some HMA proteins might sequester metals within vulnerable tissues such as the vasculature, where *HPP*/*HIPP* expression is often enriched (Mira *et al*., 2001; Barth *et al*., 2009; Tehseen *et al*., 2010; Cowan *et al*., 2018) or in subcellular structures such as plasmodesmata, ready for delivery or release as part of a strategy to enhance stress tolerance.

## Conclusion

HMA domains can be found in multiple protein families in plants, including HPPs, HIPPs, P_1B_-type ATPases, and (occasionally, as HMA-like structures) immune receptors. While the functions of the latter two families are established, many questions regarding HPP and HIPP function remain unanswered. Molecular and genetic evidence highlighted in this review suggests that HPP/HIPPs might play important roles in plant immunity, acting as both susceptibility factors and positive regulators of resistance, depending on the protein and pathosystem in question. HPPs and HIPPs have also been implicated in heavy metal homeostasis and tolerance to other abiotic stresses. Connecting these three themes could be an underlying ability for HMA proteins to directly or indirectly regulate ROS accumulation and/or modulate stress-responsive gene expression via transcription factors. A greater understanding of plant HMA biochemistry and cell biology is needed to explain their proposed roles in heavy metal tolerance. At present, it is challenging to mechanistically link observations of ion binding by HMA domains *in vitro* to *in planta* metal tolerance phenotypes.

**Fig. 3.**
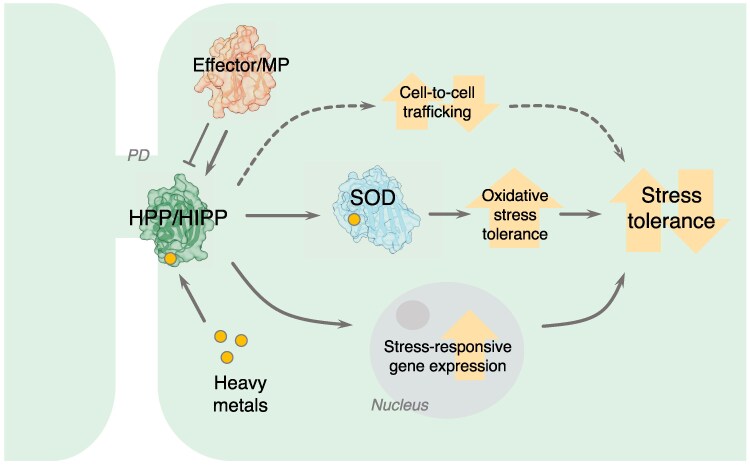
Speculative functions for plasmodesmal HMA proteins. HPP/HIPPs may be endogenous regulators of cell-to-cell trafficking and/or ROS metabolism, perhaps via provision of cofactors to metalloenzymes such as superoxide dismutase (SOD). Alternatively, they may enter the nucleus in times of stress, altering gene expression via their interaction with transcription factors. Binding of pathogen effectors or viral movement proteins (MPs) may promote or inhibit HPP/HIPP function. Dashed lines indicate hypotheses currently unsupported by empirical evidence. PD, plasmodesma.

Further study is also required to test the biological significance of HPP/HIPP plasmodesmal localization, although some speculative hypotheses are outlined in Fig. 3. Are these proteins lying in wait for effectors, viral movement proteins, or heavy metals, ready to shuttle to cell nuclei in times of stress to modify gene expression? Are they endogenous regulators of plasmodesmal aperture, targeted by pathogens to adjust cell-to-cell connectivity during infection? Do they regulate plasmodesmata-localized ROS responses via unknown interactors? In-depth studies of pathogen-targeted HPP/HIPP cell biology, including surveys of their binding partners and localization during stresses, may deepen our understanding in the future.
